# Localized gingival enlargements. A clinicopathological study of 1187 cases

**DOI:** 10.4317/medoral.22263

**Published:** 2018-04-24

**Authors:** Paris Tamiolakis, Eirini Chatzopoulou, Fiorina Frakouli, Konstantinos I. Tosios, Alexandra Sklavounou-Andrikopoulou

**Affiliations:** 1 DDS, Postgraduate Student, Department of Oral Medicine and Pathology, School of Dentistry, National and Kapodistrian University of Athens, Greece; 2 DDS, Msc in Oral Biology, School of Dentistry, National and Kapodistrian University of Athens, Greece; 3 DDS, School of Dentistry, National and Kapodistrian University of Athens, Greece; 4 DDS, PhD, Assistant Professor, Department of Oral Medicine and Pathology, School of Dentistry, National and Kapodistrian University of Athens, Greece; 5 DDS, MSc, PhD, Professor, Head of Department of Oral Medicine and Pathology, School of Dentistry , National and Kapodistrian University of Athens, Greece

## Abstract

**Background:**

To describe the incidence, demographic and clinical features of 1187 localized gingival enlargements.

**Material and Methods:**

1187 cases of localized gingival enlargements diagnosed during a 20-year period were retrospectively collected. The patients’ gender and age, as well as the main clinical features of the tumors were retrieved from the biopsy report forms.

**Results:**

The 1187 localized gingival enlargements represented 6.23% of 19.044 biopsies performed during the study period. 756 females and 427 males were affected with a mean age of 41.92±19.68 years. The lesions appeared as smooth (52.4%), granular (17.9%) or rough (13.16%) tumors, elastic (50.73%) or soft (29.56%) in consistency and red (60.8%), normal (28.58%) or white (8.17%) in color. The majority of the lesions (85.17%) were reactive in origin with pyogenic granuloma being the most common. In 1.1% of the cases a diagnosis of malignant lesion was rendered.

**Conclusions:**

All localized gingival enlargements should be submitted for microscopic examination because in approximately 1% of cases they are malignant.

** Key words:**Localized gingival enlargements, gingival mass, gingival lesions, gingival reactive lesions, gingival malignant lesions.

## Introduction

Localized gingival enlargements (LGEs) are common in clinical practice (1,2) and are usually of reactive origin (1,2) developing as a response to chronic local irritation or trauma (3). Most published studies focus on reactive LGEs (3-6) i.e. fibrous hyperplasia (FH), pyogenic granuloma (PG), peripheral ossifying fibroma (POF) and peripheral giant cell granuloma (PGCG). However, benign and malignant neoplasms (2), lesions of dysplastic origin (7) and lesions representing manifestations of systemic diseases (2) may also occur. Truschnegg *et al.* (8) and Bernick (9) studied 92 and 864 cases of LGEs, respectively. However, in the first study the number of cases was too small for valid conclusions to be made, while in the later LGEs were studied together with localized enlargements of the palate.

The aim of the present study is to report the incidence, demographic and clinical features of 1187 LGEs.

## Material and Methods

In this retrospective study all Biopsy Request Forms accompanying biopsies submitted to our Laboratory from 1995 to 2015 were searched using the keywords “tumor” and “gingiva” in the predefined list of clinical terms. For each case the patient’s gender and age, location and clinical features of the lesions were tabulated. Final diagnosis was retrieved from the Pathology report. Cases reported as localized on the “alveolar mucosa” or “edentulous alveolar ridge” were excluded from the study.

## Results

A total of 1187 cases of LGEs affecting 1187 patients represented 6.23% of 19.044 biopsies accessioned during the study period. Diagnoses are tabulated in  Table 1. Most lesions (85.17%) were reactive in origin, with PG being the most common (27.8%), followed by POF (27.38%), FH (13.73%), PGCG (12.38%) and fibroepithelial hyperplasia (FEH) (3.88%). Other common lesions in descending order were papilloma (2.27%), gingival inflammation (2.11%) giant cell fibroma (1.94%) and peripheral odontogenic tumors (1.43%). Malignant lesions were rare (1.1%).

**Table 1:**
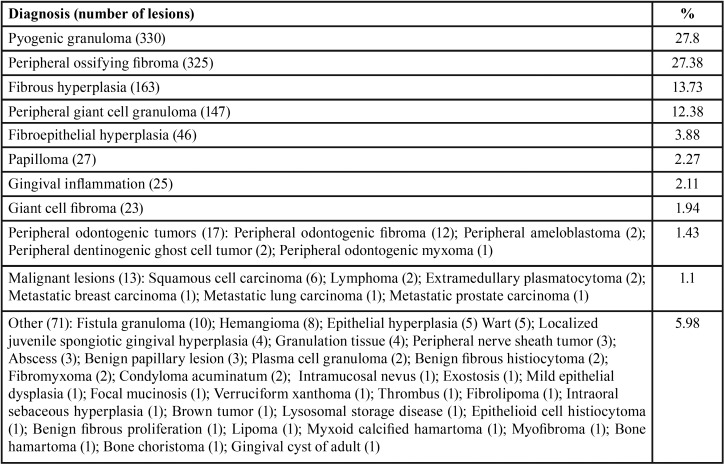
Diagnosis of localized gingival enlargements.

**Figure 1:**
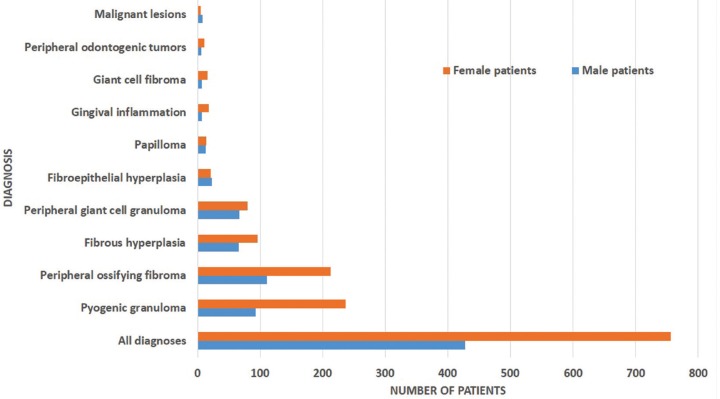
Gender of patients (for lesions occurring at least in 10 patients).

**Figure 2:**
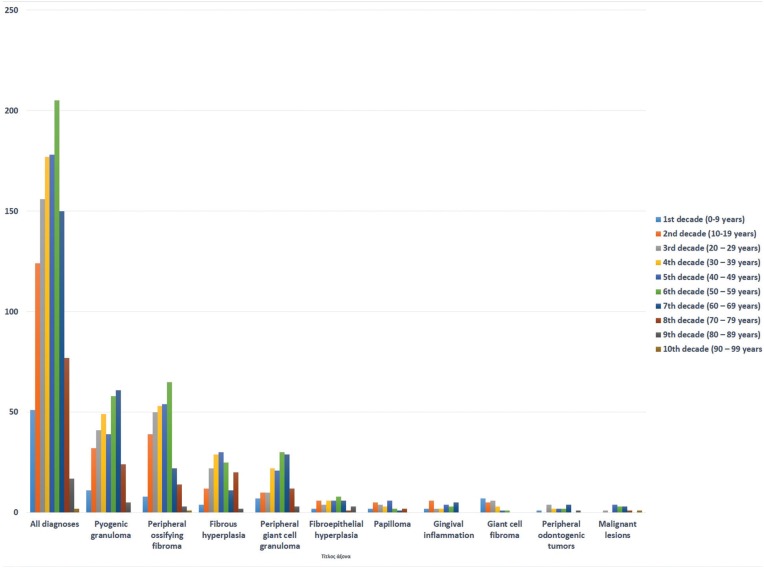
Age of patients related to decade of life (for lesions occurring at least in 10 patients).

**Figure 3:**
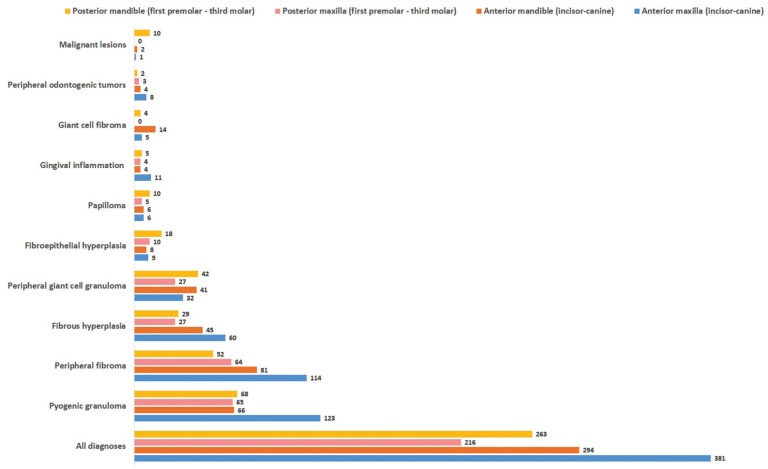
Site of localized gingival enlargements (for lesions occurring at least in 10 patients).

In Figures 1 and 2 gender and age distribution for all LGEs as well as for particular entities diagnosed in at least 10 patients are tabulated. As a whοle, LGEs showed a female predominance, with 756 cases (63.91%) occurring in female patients compared to 427 (36.09%) in males (male to female ratio 0.56:1). Most patients were in the 4th – 6th decade of life, with a mean age of 41.92±19.68 years (median age 43 years). The difference between the mean age of males (41.12±21.2 years, median age 42 years) and females (42.4±18.76 years, median age 43 years) was not statistical significant. Giant cell fibroma and papilloma occurred in younger patients and malignant lesions in older patients, compared to LGEs. LGEs showed a slight predilection for the maxilla (51.73%), compared to the mandible (48.28%), as well as for anterior areas (58.49%), compared to posterior ones (41.51%). They were more common in the anterior maxilla (33.02%), followed by the anterior mandible (25.48%), posterior mandible (22.79%) and posterior maxilla (18.7%). FEH, papilloma and malignant lesions occurred more frequently in the posterior part of the mandible (Fig. 3).

Clinically LGEs were described as sessile (50.9%) or pediculated masses (49.1%); of red (60.8%), normal (28.58%) or white (8.17%) color; elastic (50.73%) or soft (29.56%) in consistency; and with smooth (52.4%), granular (17.9%) or rough (13.16%) surface. There were few significant divergences from the aforementioned clinical characteristics. Concerning pediculus, lesions that differed were gingival inflammation, peripheral odontogenic tumors and malignant lesions, the vast majority of which were sessile (77.27%, 100% and 88.89% respectively). The majority of PG, PGCG, and gingival inflammation (85.13%, 84.78% and 91.3% respectively) were red in color, whereas 37.5 % of papillomas and 52.38% of giant cell fibromas were white. In contrast to all the other LGEs, papillomas (41.67%) and malignant lesions (25%) most commonly had a granular rather than smooth surface. Also papillomas (29.17% of cases) and FEH (17.5% of cases) were more possible to have a papillary surface, while malignant lesions (25% of cases) and giant cell fibromas (23.81% of cases) to have a rough one. LGEs measured approximately 1.15±0.8cm (median size 1cm) in the largest diameter. Malignant lesions had the largest mean size among LGEs (1.98±0.84cm.), while papillomas (0.58±0.31 cm) and giant cell fibromas (0.63±0.35cm) the lowest. LGEs were asymptomatic in 82.39% of cases justifying the long duration of 12.76±21.61 months (median duration 5 months) before diagnosis. FH (20.33±32.84 months) and giant cell fibroma (33.53±39.48 months) had the longest mean duration before diagnosis, whereas malignant lesions (2.64±1.98 months) and gingival inflammation (6.12±9.23 months) had the shortest.

Recurrence was reported in POF (7 cases), PG (5 cases), PGCG (4 cases), FH (2 cases) papilloma and peripheral odontogenic fibroma (1 case each).

## Discussion

In this retrospective study the incidence, demographic and clinical features of 1187 LGEs were described, while in previous studies only reactive LGEs were included (3-6). They represented 6.23% of all biopsies accessioned in the study period which is higher than the percentage of 4% seen in adults (10) and 2.3% in children up to 16 year-old (11) reported in other studies during a 30-year period.

Most LGEs, PG, POF, FH and PGCG were of reactive origin, and represented 5.35% of all biopsies of the study period which is comparable to the frequency reported in previous studies, 6.7% (3), 6.4% (4), 5% (5) and 3.6% (6) respectively. Although in the aforementioned studies FH was the most common reactive LGE, representing 31.8% (3), 61.2% (4), 45.5% (5), and 61% (6), in the present study PG was the most common (34.2%), followed by POF (33.68%) and FH (16.9%). Differences in the relative frequency of reactive LGEs in various studies (3-6) have been attributed to heterogeneity of the study populations or to different histopathologic criteria (3). The clinical features of the four reactive LGEs are in accordance with previous reports (3). In 18 cases of reactive LGEs recurrence was recorded, but as the data of the present study were extracted from biopsy report forms, conclusions on recurrence rates cannot be drawn.

Peripheral odontogenic tumors represented 1.43% of all LGEs and 0.09% of all specimens submitted for histopathologic examination during the study period, while in previous studies these tumors represented 0.05% of all specimens submitted for histopathologic examination (13), and their relative frequency among all odontogenic tumors ranged from 0.1% to 8.9% (13). Peripheral odontogenic fibroma was the most common peripheral odontogenic tumor followed by peripheral ameloblastoma which is in accordance with previous studies (13-15). Similarly, according to a previous report, peripheral odontogenic fibroma in our study, exhibited a female predominance, a predilection for middle aged patients, and for the anterior part of the maxilla and the mandible (16). Peripheral ameloblastoma represents 0-10% of all ameloblastomas (13), while the two cases of peripheral dentinogenic ghost cell tumors and the single case of peripheral odontogenic myxoma detected in our series are extremely unusual tumors, as only 50 cases (17) and 6 cases (18), respectively, have been included in recent reviews.

Squamous cell carcinoma was the most common malignant LGE in our study. It presented in four female and two male patients, with a mean age of 58.3 years. All lesions arose on the mandibular gingiva, five on the posterior and one on the anterior. Gingival squamous cell carcinoma accounts for less than 10% to as high as 30% of all oral squamous cell carcinomas and shows almost equal gender distribution and a predilection for older patients (19). It occurs most commonly on the posterior mandibular gingiva as an exophytic mass, usually ulcerated (19). It is noteworthy that many practitioners report reactive gingival lesions in the differential diagnosis of gingival squamous cell carcinoma (19), while in our study, a provisional diagnosis of reactive lesion was given in half of the cases of squamous cell carcinoma.

Three cases of metastatic tumors, two cases of lymphoma and two cases of extramedullary plasmatocytomas presenting as LGE were also detected in our material. The gingiva is the most common soft tissue site of metastasis (20), and the 4th most common site of oral lymphoma development followed by the maxilla, palate and the mandible (21,22). The lesions may mimic reactive lesions (20). Metastatic tumors are seen in the presence of widespread disease, and lung, breast and kidney carcinoma metastasize more often to the oral soft tissues compared to other tumors (20). In our material, the metastatic tumors originated from the breast, lung and prostate; they occurred in two male and one female patients with a mean age 64 years; two lesions presented in the posterior mandibular gingiva and the other in the anterior maxillary gingiva.

## Conclusions

LGE are common in clinical practice and most are of reactive origin, but approximately 1% are malignant. Therefore, all LGEs should be submitted for microscopic examination and not diagnosed as benign based on the clinical impression and expertise.
